# Rapid In Situ Detection of THC and CBD in *Cannabis sativa* L. by 1064 nm Raman Spectroscopy

**DOI:** 10.1021/acs.analchem.2c01629

**Published:** 2022-07-18

**Authors:** Stefania Porcu, Enrica Tuveri, Marco Palanca, Claudia Melis, Ignazio Macellaro La Franca, Jessica Satta, Daniele Chiriu, Carlo Maria Carbonaro, Pierluigi Cortis, Antonio De Agostini, Pier Carlo Ricci

**Affiliations:** † Department of Physics, University of Cagliari, S.p. no. 8 Km 0700, 09042 Monserrato, CA, Italy; ‡ Scientific Investigation Department (RIS) of Cagliari, Via Ludovico Ariosto, 24, 09129 Cagliari, CA, Italy; § Department of Life and Environmental Sciences, University of Cagliari, Via Sant’Ignazio 13, 09123 Cagliari, CA, Italy

## Abstract

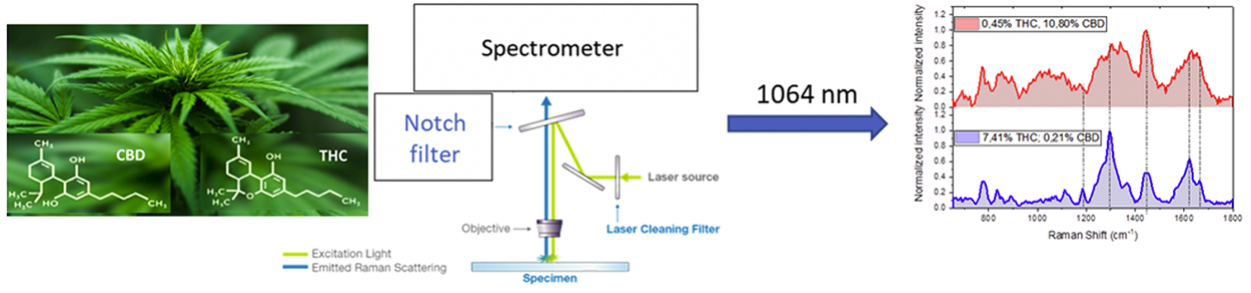

The need to find a rapid and worthwhile technique for the in situ detection of the content of delta-9-tetrahydrocannabinol (THC) and cannabidiol (CBD) in *Cannabis sativa* L. is an ever-increasing problem in the forensic field. Among all the techniques for the detection of cannabinoids, Raman spectroscopy can be identified as the most cost-effective, fast, noninvasive, and nondestructive. In this study, 42 different samples were analyzed using Raman spectroscopy with 1064 nm excitation wavelength. The use of an IR wavelength laser showed the possibility to clearly identify THC and CBD in fresh samples, without any further processing, knocking out the contribution of the fluorescence generated by visible and near-IR sources. The results allow assigning all the Raman features in THC- and CBD-rich natural samples. The multivariate analysis underlines the high reproducibility of the spectra and the possibility to distinguish immediately the Raman spectra of the two cannabinoid species. Furthermore, the ratio between the Raman bands at 1295/1440 and 1623/1663 cm^–1^ is identified as an immediate test parameter to evaluate the THC content in the samples.

## Introduction

*Cannabis sativa* L. is known for being a class of plants with a wide variety of derived products from food and textile fiber to psychotropic substances.^1−3^

In the 60s, cannabinoids have been classified as the main biological active components of the *Cannabis* plants.

In the past, the term cannabinoids referred to a group of compounds with a typical C_21_ structure present in *C. sativa*. The modern definition, with greater emphasis on chemistry and pharmacology, includes instead all those molecular structures that interact with cannabinoid receptors.^4^ This new classification has created numerous subcategories that take into account the origin of the compound and its synthetic or natural origin. In the common nomenclature, the term “phytocannabinoid” is used for natural plant compounds, with the term “endocannabinoids” used for the endogenous ligands of cannabinoid receptors. The synthetic agonists of these receptors have been classified according to their degree of pharmacological proximity with phytocannabinoids, which are divided into “classic” vs “nonclassic”.^5,6^

Over 60 cannabinoids are present in *Cannabis*, most of which belong to the subclasses of the cannabigerol (CBG), cannabichromene (CBC), cannabidiol (CBD), and delta-9-tetrahydrocannabinol (Δ9-THC) types.^4,7−9^

**Scheme 1:**
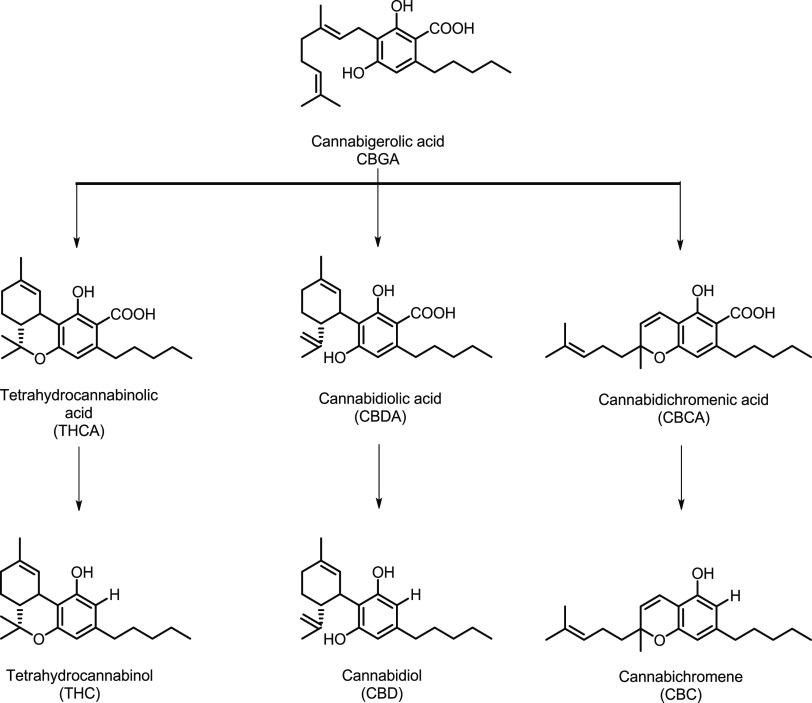
Subclasses of Cannabinoids Mainly Present in *Cannabis* Plants

While THC is a psychoactive and often illicit drug, CBD and CBG are legal substances that have a variety of pharmacological properties such as reducing chronic pain, inflammation, anxiety, and depression. All the cannabinoids are biosynthesized from the cannabigerolic acid that is present in the plants in a range of concentrations of 25–35% w/w, Scheme 1.

The amount of the ratio of THC/CBD divides *Cannabis* plants into three chemotypes. A ratio bigger than 1 is a characteristic of drug plants (chemotype I), while a ratio near 1 or smaller is a characteristic of chemotypes II and III of intermediate-type plants and fiber-type plants, respectively.

Over the past decade, substantial efforts have been made to develop *Cannabis* strains capable of producing large quantities of CBD and CBG. Ideally, these plant varieties should produce a very limited amount of THC, and usually, it must be below 0.3%. This threshold is applied by different European states and USA to distinguish between legal and illicit hemp/*Cannabis*.^10,11^

The consumption of psychoactive substances is continuously increasing, and the European Monitoring Centre for Drugs and Drug Addiction (EMCDDA) reports a 25% increase, in the last 25 years, of the illicit market.^12^ For this reason, it is essential to explore fast and valuable ways of identifying these psychoactive substances.^13−17^

Different techniques are used to determine the amount of cannabinoids in *Cannabis*: high-performance liquid chromatography (HPLC) and gas chromatography (GC) combined with both a flame ionization detector (FID) and mass spectrometer (MS).^18−21^

The analysis using these techniques involves a pretreatment of the samples. Most of the time, extraction methods are used, and the choice of the extraction method depends on the nature of the starting material. In addition to traditional methods such as maceration, distillation, or boiling, many other modern extraction methods and techniques can be applied for the extraction of natural cannabinoids. These methods include Soxhlet, accelerated solvent extraction, pressurized liquid extraction (PLE), microwave-assisted extraction (MAE), ultrasonic-assisted extraction (UAE), supercritical fluid extraction (SFE), solid-phase extraction (SPE), and solid-phase microextraction (MSPE).^22−26^ It is worthy of importance that all the aforementioned methods are not portable and destructive and often require long processing times.

Raman spectroscopy can be selected as a valuable and cost-effective technique for the detection of cannabinoids. In the literature, few attempts have been made to introduce Raman spectroscopy for the identification and quantification of phytocannabinoids.^13,27,28^ The use of such a technique is often hampered by the high fluorescence generated by the excitation wavelength that overlaps the Raman signal.

Recently, Sanchez et al.^29^ proposed the use of NIR–Raman spectroscopy (RS) for confirmatory, noninvasive, and nondestructive differentiation between hemp and *Cannabis*. They showed the results obtained using an 830 nm lasers source, 10s acquisition time, and 495 mW power for the analysis of the extracted oil from different types of *Cannabis*. This method requires pretreatment of the samples, which includes an extraction procedure using organic solvents and requires the use of a high-power laser for the Raman measurements.

Herein, we present that using 1064 nm Raman spectroscopy, it is possible to detect the cannabinoids focalizing the measurement in the glandular trichomes of the plant that appear as reddish circular bubbles in the inflorescence. The investigated samples, coming from the Scientific group of Scientific Investigation Department (RIS) of Cagliari (Italy), were analyzed without a previous treatment. Each sample was observed using an optical microscope, and the spectra were acquired at several points to verify the homogeneity. The multivariate analysis was applied to verify the reproducibility of the spectra and to prove the possibility to rapidly distinguish the Raman spectra of THC and CBD.

## Experimental Section

### Materials

*Cannabis* samples have been provided by the Scientific Investigation Department (RIS) of Cagliari.

A set of 42 samples with different amounts of THC and CBD was studied. The samples come from different plantations seized from the Scientific Investigation Department (RIS) of Cagliari for the investigation of the content of cannabinoids.

GC-FID analysis has been performed at the Scientific Investigation Department. For the analysis, a methanolic solution of each sample has been prepared, and androsterone (purchased from Sigma-Aldrich) has been used as an internal standard.

For the Raman measurements, performed at the Physics department of the University of Cagliari, all the samples have been analyzed without a previous treatment, and the spectra were collected from the leaves and inflorescences. Different regions (glandular trichome-rich, inflorescence, leaves, and stems) has been distinguished using a microscope (objective 20×). For each sample, at least eight different points were analyzed, and the average of them was considered as a representative.

### Characterization Techniques

For GC-FID analysis, an Agilent Technologies 7890 series gas chromatograph (GC) was employed, equipped with a split injector and an ULTRA 2 fused silica column: 5% phenyl-methylpolysiloxane, 20 m × 0.32 mm i.d., film thickness of 0.52 μm. The analysis has been performed under isothermal conditions at 240 °C for 16 min. The injector was maintained at 290 °C. Helium was the carrier gas at 1.4 mL/min; the sample (1 μL) was injected in the split mode (1:50). The GC was fitted with a FID, model 7890B. FID conditions were set as follows: temperature was 300 °C; hydrogen was the carrier gas at 40 mL/min; and nitrogen was the makeup gas at 25 mL/min.

Raman spectra were acquired in the back scattering geometry with excitation wavelength at 1064 nm generated using a Nd:YAG laser, to avoid luminescence contribution in the visible range. The system operates in the Stokes region up to 2500 cm^–1^. Measurements were performed in air at room temperature with a BWTEK i-Raman ex spectrometer with a spectral resolution of 9 cm^–1^.

Samples of air-dried *C. sativa* L. bracteal leaves from inflorescences of both legal and illegal chemotypes were observed by optical microscopy to better interpret Raman microscopy results. A stereomicroscope equipped with the HD cam TiEsseLab TrueChrome HD IIS (Tiesselab, Italy) and software TiEsseLab IS CAPTURE Rel. 3.6.7 (Tiesselab, Italy) was used to take measurements on the structures of interest and to capture the related images. A compound microscope was also used to observe in the bright field and magnify up to 400× the structures of interest.

Multivariate analysis was used for the statistical study of the collected data. All the collected spectra were analyzed in a range that includes wavenumbers from 655 to 1800 cm^–1^, and then, the data were normalized from 0 to 1 for the direct comparison.

## Results and Discussion

**Figure 1:**
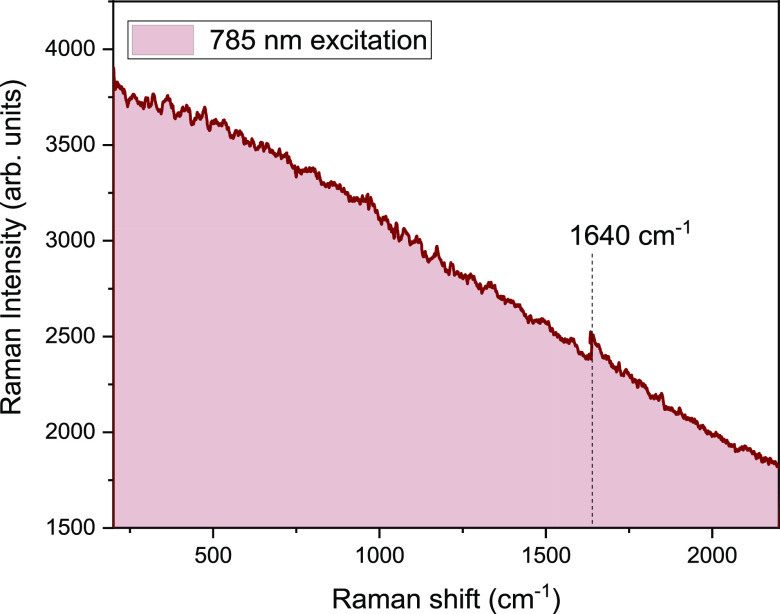
Raman spectrum of fresh inflorescence with 785 nm excitation wavelength.

The Raman spectrum of a natural plant is often very difficult to acquire due to the high fluorescence generated by chlorophyll B and carotenoids. Figure 1 reports the spectrum obtained by exciting the *Cannabis* using a near-infrared laser (785 nm). A very broad luminescence overlaps the part of the spectrum that contains the Raman signal, obstructing clear information about the presence of cannabinoid species. On the other hands, the use of a 1064 nm laser as an excitation source allows collecting very clear spectra and underlining the difference in terms of vibrational modes of the two cannabinoids.

**Figure 2:**
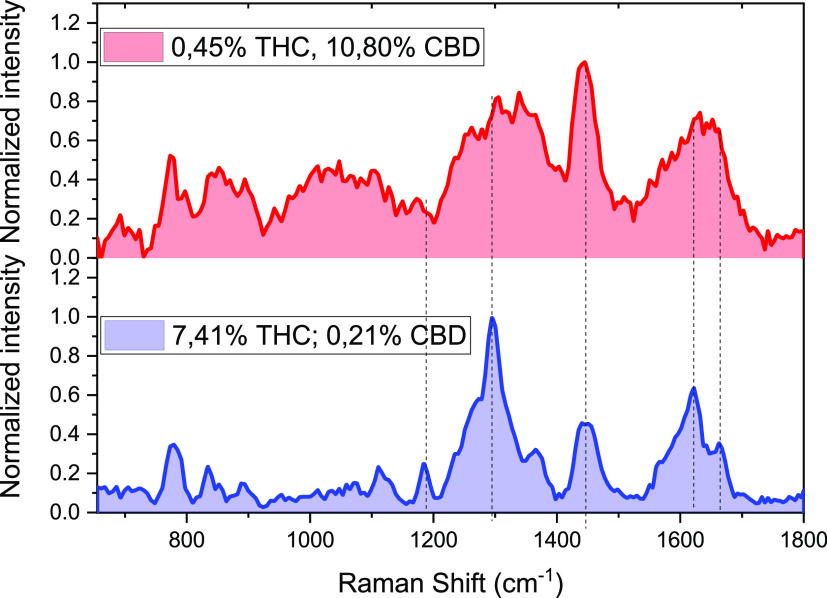
Raman spectra of CBD- and THC-rich plants, collected using 1064 nm laser excitation (sample 6 and sample 26, respectively in Table 1).

Figure 2 shows the experimental Raman spectra, collected using 1064 nm laser excitation, of CBD (red)- and THC (blue)-rich plants. The quantitative analysis for both CBD and THC in the samples was obtained by GC-FID measurements (see the Supporting Information) using androstane as an internal standard. The exact composition is reported in Figure 2 and in Table 1 (sample 6 and sample 26). The spectra were collected without a previous treatment of the sample, directly on the inflorescence, overcoming the extraction procedure from the plants and/or post-processing of the experimental data.

**Table 1:** GC-FID Results of the Analyzed Samples with the Corresponding Amounts of THC and CBD

sample	% THC	% CBD
1	1.60%	0.30%
2	1.90%	0.27%
3	6.90%	0.30%
4	7.78%	0.25%
5	10.49%	0.29%
6	7.41%	0.21%
7	6.42%	0.24%
8	6.42%	0.24%
9	8.42%	0.27%
10	6.23%	0.26%
11	8.61%	0.22%
12	5.80%	0.21%
13	4.14%	0.21%
14	3.61%	0.29%
15	2.02%	0.09%
16	4.34%	0.13%
17	3.68%	0.17%
18	4.12%	0.13%
19	3.01%	0.11%
20	3.35%	0.11%
21	3.56%	0.10%
22	5.11%	0.18%
23	4.15%	0.15%
24	3.20%	0.17%
25	7.49%	0.24%
26	0.45%	10.80%
27	0.45%	10.80%
28	0.26%	5.22%
29	0.29%	5.48%
30	0.19%	3.53%
31	0.33%	6.26%
32	0.33%	6.53%
33	0.23%	4.54%
34	2.87	5.69
35	0.55	6.49
36	0.60	8.61
37	1.28	13.80
38	1.58	14.07
39	0.99	10.89
40	1.45	12.46
41	1.30	10.62
42	0.90	9.51

**Figure 3:**
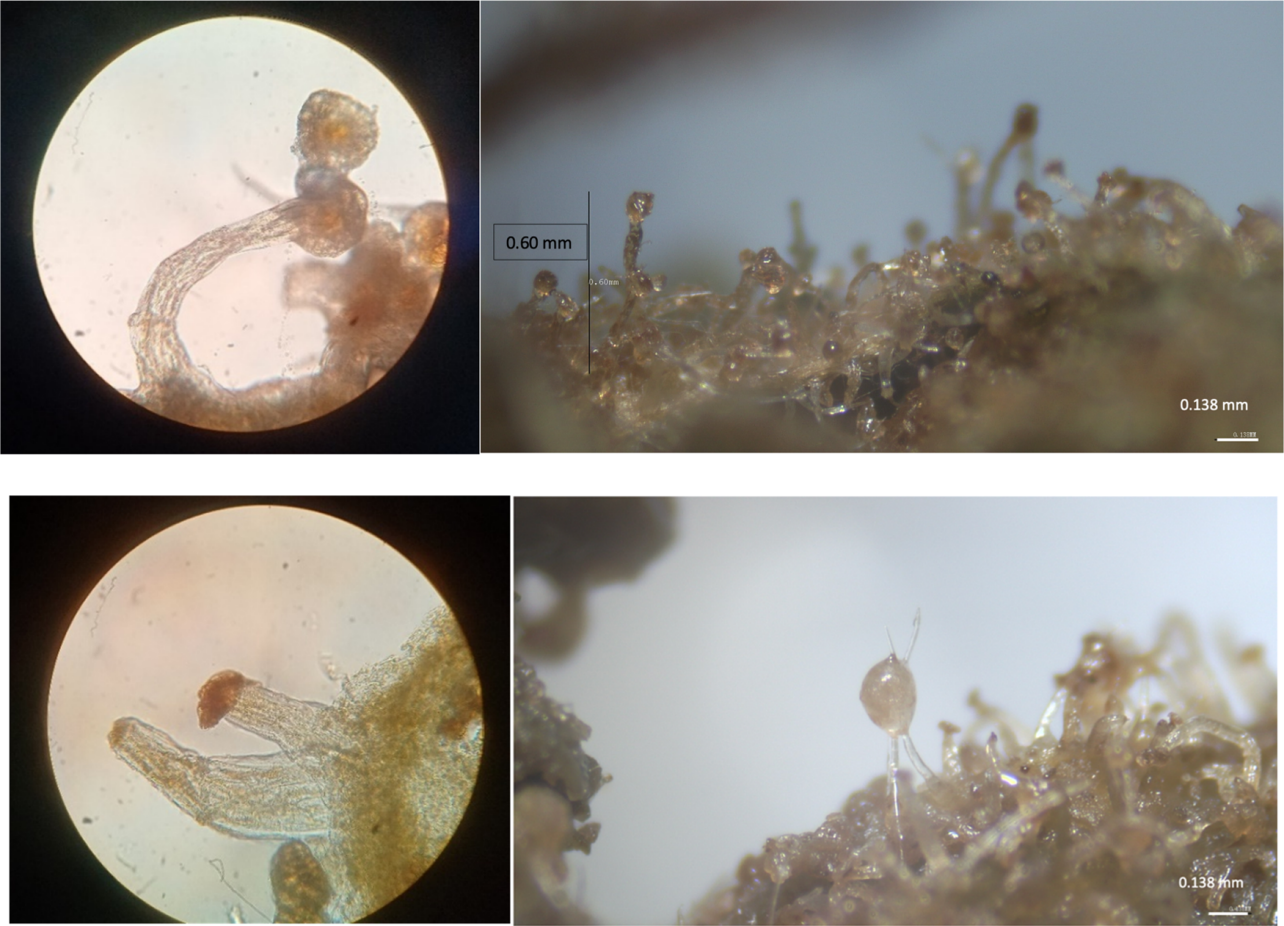
Optical microscopy images of CBD- and THC-rich plants.

Both the samples present several vibrational bands between 780 and 1600 cm^–1^ due to the similarity in terms of the chemical structure of THC and CBD (Scheme 1). However, most of the peaks present very similar spectral shifts but with substantial differences from a more accurate analysis.

The main vibrational bands of the THC-rich plant are observed at 775, 780, 835, 1185, 1295, 1321, 1365, 1570, 1600, 1623, and 1666 cm^–1^, while in CBD-rich plants, vibrational bands at 775, 865, 985, 1012, 1080, 1104, 1302, 1340, 1370, 1437, 1643, and 1663 cm^–1^ were observed.^29,30^ The assignment of these bands is reported in Table S1 (Supporting Information).

The Raman spectrum of the THC-rich plant exhibits a prominent vibrational band at 1295 cm^–1^, which in the spectrum of the CBD-rich plant is not well-pronounced. In addition THC shows a peak located at 1185 cm^–1^ not observed in CBD.^29^ A substantial difference is also observed in the region of the aromatic vibrations, where a vibrational band located at 1623 cm^–1^ is representative only of the THC-rich plant, while a very intense vibrational band at 1440 cm^–1^ reflects the presence of a high amount of CBD in the sample.

The structures with the Raman information on the cannabinoid’s composition appeared as densely colored circles of sub-millimetric dimensions. Optical microscopy allowed us to identify said structures as glandular trichomes densely covering the observed bracteal leaves in both legal and illegal chemotypes. (Figure 3 and SI) The observed samples presented glandular and simple trichomes, the former producing the substances observed by the Raman microscopy approach. Optical microscopy alone was not able to discriminate legal and illegal chemotypes in the observed samples.^32^

**Figure 4:**
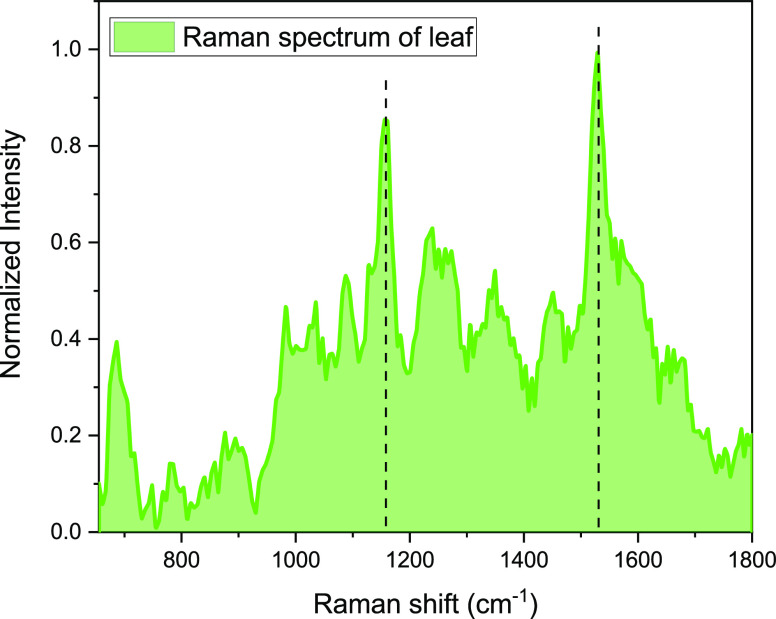
Raman spectrum of leaves collected using 1064 nm laser excitation.

Glandular trichomes are a widespread structure in the plant kingdom performing a variety of tasks in plants (*e.g.,*, resistance to abiotic stressors, protection against herbivory, and defense against pathogens). The morphology of these structures could vary a lot among different species and within the same species/individuals too. *Cannabis sativa* L. is featured by the presence of stalked glandular trichomes producing and storing the substances (mainly secondary metabolites and terpenes) of economic and legal interest (Figure 4).^33−36^ It is worth noting that the spectra are not uniform in all the parts of the plant. Different regions can be easily distinguished by means of the objective of the microscope (20×). The Raman features reported and discussed in the previous section come from the glandular trichome-rich parts of the inflorescence (*e.g.*, bracteal leaves, inflorescence axis, etc.), while the Raman spectra obtained from the other parts of the plants (leaves and stems) (Figure 4) do not give deep information. The main features at 1155 and 1525 cm^–1^ are related to cellulose and carotenoids, respectively, while no clear bands from cannabinoids are detected regardless of the cannabinoid species.^31^

In this work, 42 different plants were analyzed using Raman spectroscopy, and GC-FID spectroscopy was used to confirm the results. From each plant, at least four different points were analyzed, and the average of them was considered as a representative. Then, the samples were divided in two families: THC-rich samples (in blue in Table 1) and CBD-rich samples (in red in Table 1). The multivariate analysis was performed separately for each group. The spectra were normalized and then truncated to include wavenumbers in the range of 655–1800 cm^–1^ and scaled to unit variance via standard normal variate correction to give all spectral regions equal importance.

In this analysis, the principal component allows us to examine the relationship of the data. Each principal component represents the linear combination of the variables and gives a maximized variance. In this case, the variables to be considered are the maximum and minimum points of the spectrum or, in other words, the spectra themselves. It follows that the main components of the multivariate analysis represent the spectrum “common” to the various samples, and the coefficients represent how similar the spectra obtained from the various samples are.

The principal component has a variance of approximately 84%, and the second component has a variance of approximately 10% (eigenvalues of 7.83 and 1.17, respectively).

**Figure 5:**
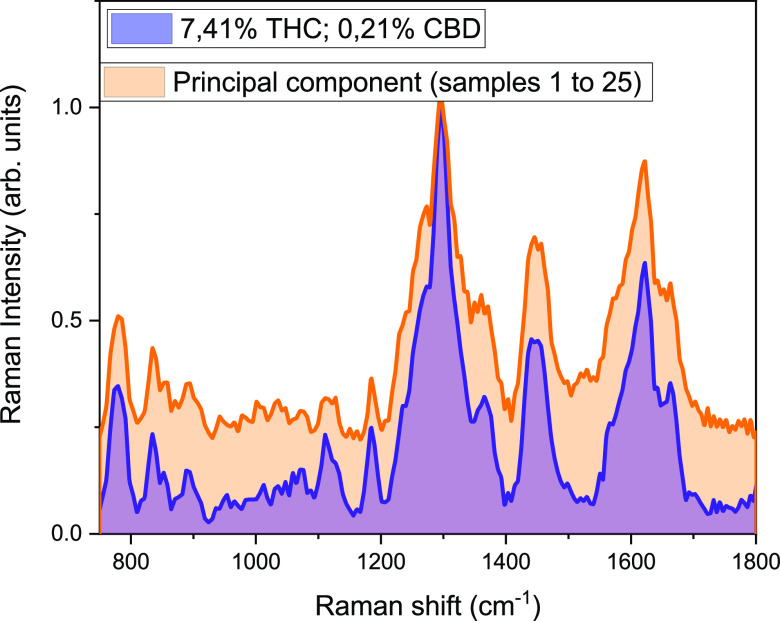
Principal component for the THC-rich plants compared with a single spectrum of a plant of the same family.

**Figure 6:**
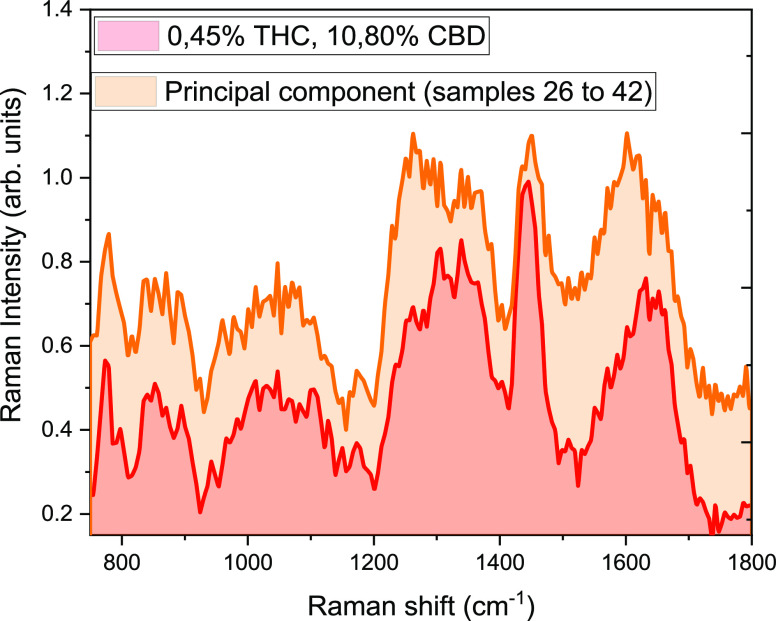
Principal component for the CBD-rich plants compared with a single spectrum of a plant of the same family.

Figures 5 and 6 show the principal components of each family (THC- and CBD-rich, respectively) compared with the single spectrum from the fresh plants. The similarity between the principal component and the spectrum acquired in the plant underlines the high reproducibility of the experimental data and the possibility to perform the analysis without the use of statistical treatment of the acquired spectra.

**Figure 7:**
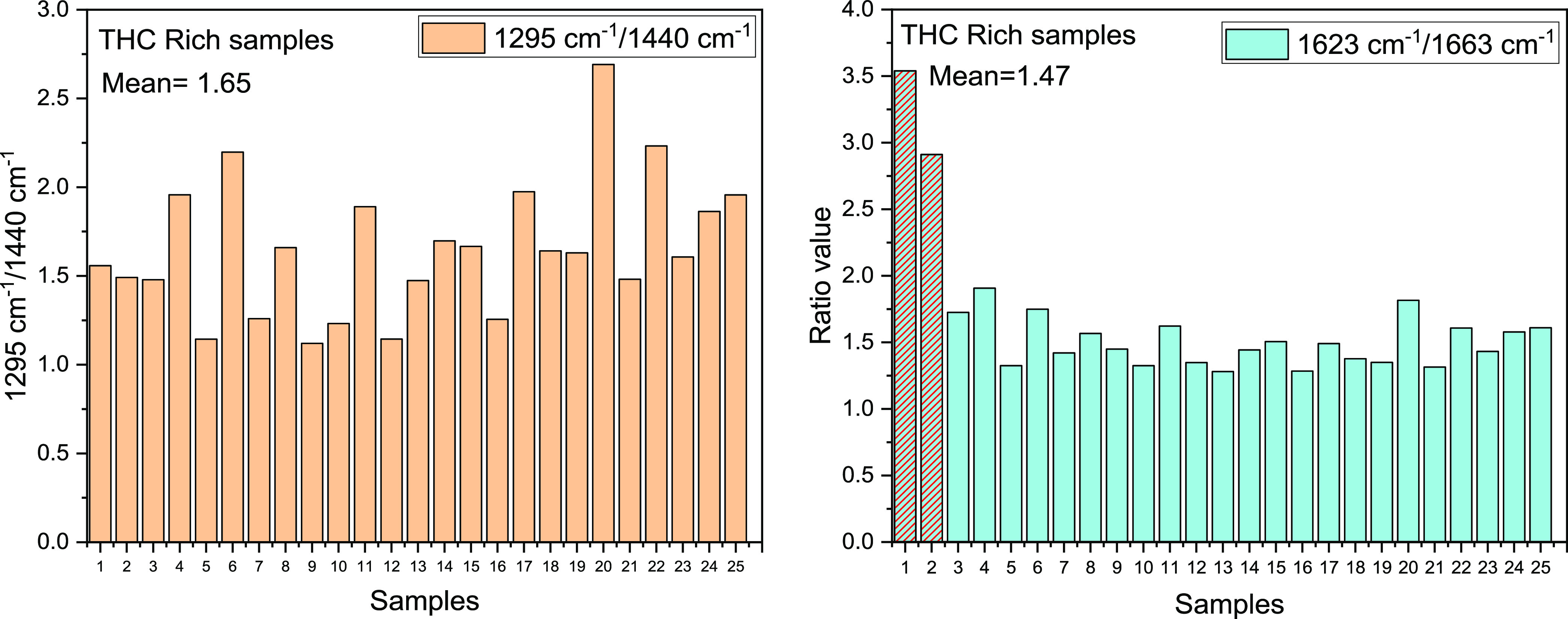
Mean of the ratio between 1295/1440 and 1623/1663 cm^–1^ peaks for the THC-rich samples.

**Figure 8:**
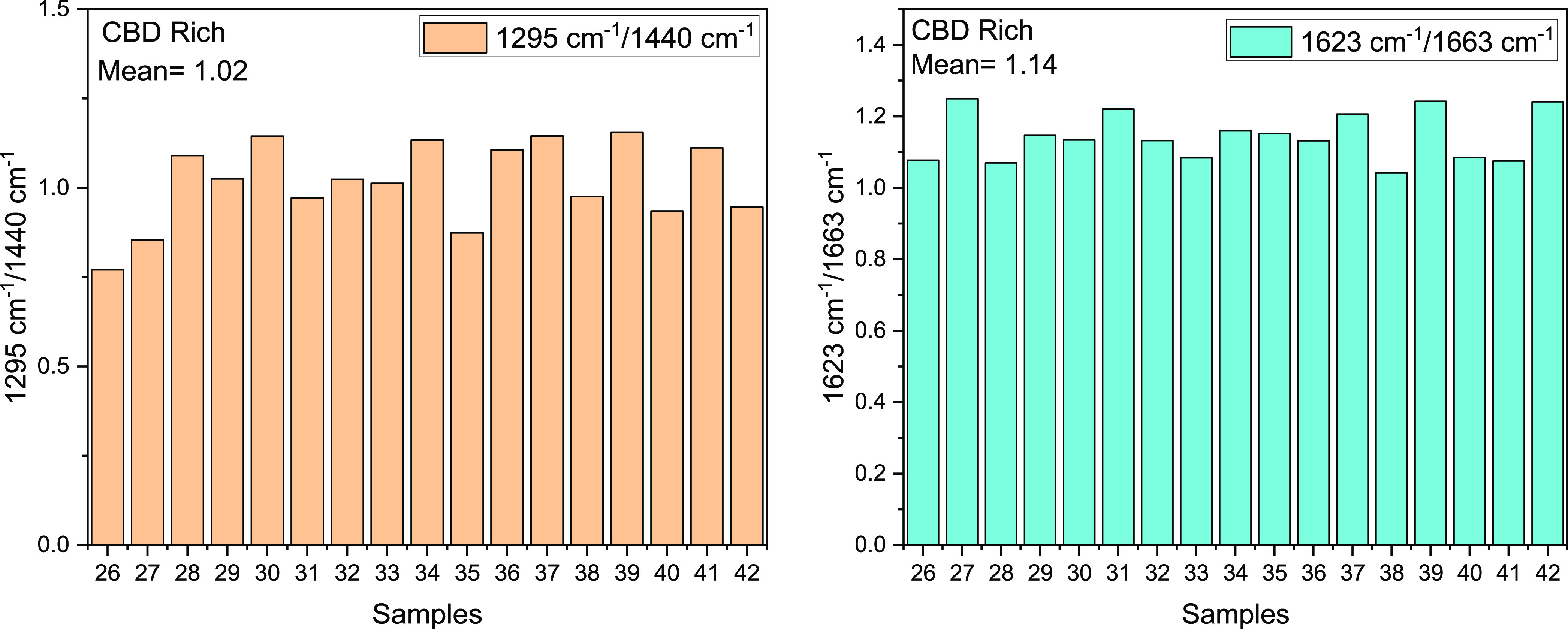
Mean of the ratio between 1295/1440 and 1623/1663 cm^–1^ peaks for the CBD-rich samples.

The deeper analysis of the spectra could give some analytical parameters for the evaluation of the cannabinoid content. The main peaks of the two groups are related to the C-C-H bending vibration of the aliphatic chain of the THC at 1295 cm^–1^ and the CH_2_ vibration at 1440 cm^–1^ for the CBD-rich sample.^37^ It is worth analyzing the ratio between the peak at 1295 cm^–1^ and the peak at 1440 cm^–1^. Second, the ratio between the peak at 1623 cm^–1^ and the one at 1663 cm^–1^ can give important insights into the presence of the two cannabinoids. (Figures 7 and 8)

The analysis showed a clear distinction in the two families, THC-rich samples showing always higher ratio values as compared to the ones of CBD-rich samples, with mean values considerably higher (1.65 and 1.47, for the THC-rich samples and 1.02 and 1.14 in the CBD-rich plants).

**Figure 9:**
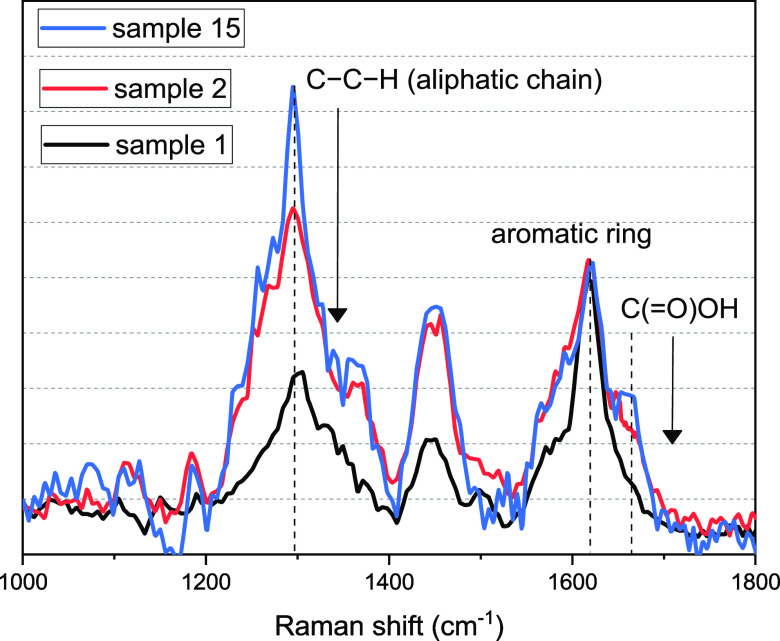
Comparison of Raman spectra of ground mixed sample and fresh inflorescence.

The analytical treatment of the experimental data points out the anomalous behavior of the Raman spectra of samples 1 and 2 with respect to all the other peaks and more in particular with sample 15. These samples have a close content of THC, but the ratio between the aromatic chain vibrations at 1623 and 1663 cm^–1^ is relatively higher than that of all the other samples. However, it is important to underline that the samples 1 and 2 are mixed ground dry samples, and the variation in the ratio is connected to the abrupt decrease of the peak at 1663 cm^–1^, assigned to the vibration of the carboxylic group of the THCA.^38^ We argue that this variation is connected to the conversion of the THCA to THC (see Scheme 1) generated by the temperature increase. On the other hand, sample 15 (coming from fresh inflorescence) still presents the THCA contribution (Figure 9).

Furthermore, the dry samples present a lower relative contribution of the band at 1295 cm^–1^, related to the fragmentation of the aliphatic chains and damages due to the mechanic process.

We propose that, due to shredding, the fragile glandular trichome heads are broken, and their content sticks out, and consequently, it is subjected to a series of chemical and physical environmental stress factors (light radiation, atmospheric oxygen, temperature variation) that lead it to degrade compared to that contained in the “protected” environment of the intact trichome heads of the fresh samples (Figure 9).

More specific analysis on the external effect on cannabinoids is mandatory to confirm the hypothesis and to produce new evidence on the Raman spectra.

## Conclusions

The Raman spectra of two different families of *Cannabis*, THC- and CBD-rich, have been acquired, and all the main Raman modes were assigned. The spectra were acquired on 42 natural samples with excitation wavelength at 1064 cm^–1^. Furthermore, with respect to previous studies, we demonstrate the possibility to quickly distinguish the spectra between the two cannabinoids without any data processing, also thanks to the absence of natural fluorescence.

Multivariate analysis underlines the high reproducibility of the experimental data on natural samples, but it is shown that it was not necessary to distinguish the chemotype of the plants.

The analytical signatures of THC and CBD Raman spectra were identified in the ratio between the peaks at 1295 and 1440 cm^–1^ and between 1623 and 1663 cm^–1^, whereas higher values of the ratios have been clearly observed for the THC-rich samples.

Finally, a preliminary analysis on dry ground samples and fresh inflorescence evidences some interesting insights into the Raman spectra. The decrease of the peak at 1663 cm^–1^ in dry ground samples is related to the total conversion of THCA into THC (because of the grinding-induced temperature effect), while the relative decrease of the band at 1250 cm^–1^, assigned to aliphatic chain vibrations, is proposed to be related to the physical environmental stress generated by the grinding.

In summary, we proved the high potential of Raman spectroscopy in the IR region, where the absence of fluorescence permits us to acquire and analyze the experimental data directly on fresh plants. Therefore, Raman spectroscopy is indeed highly proposed as a fast and reliable tool for the identification of illegal items and/or for the evaluation of the cannabinoid content in fresh plants.

## Supporting Information Available

GC-FID results for THC-rich samples and for CBD-rich samples; optical images obtained with optical microscopy of THC- and CBD-rich plants; and assignation of the Raman modes in THC- and CBD-rich plants (PDF)
