# Supplementary material for: Rapid In Situ Detection of THC and CBD in Cannabis sativa L. by 1064 nm Raman Spectroscopy
Source: Anal Chem. 2022 Jul 18;94(29):10435–42. doi: 10.1021/acs.analchem.2c01629 (PMC9330313; doi:10.1021/acs.analchem.2c01629)
Supplement: Supplementary file 1 — ac2c01629_si_001.pdf [file ac2c01629_si_001.pdf]

Supporting information for:

Rapid in-situ detection of THC and CBD in *Cannabis sativa* L. by 1064 nm Raman spectroscopy

Stefania Porcu[a*], Enrica Tuveri[b], Marco Palanca[b], Claudia Melis[b], Ignazio Macellaro La Franca[b], Jessica Satta[a], Daniele Chiriu[a], Carlo Maria Carbonaro[a], Pierluigi Cortis[c], Antonio De Agostini[c], Pier Carlo Ricci[a*]

[a] Department of Physics, University of Cagliari, S.p. no. 8 Km 0700, 09042 Monserrato, CA, Italy; stefania.porcu@dsf.unica.it; carlo.ricci@dsf.unica.it ; jessica.satta@dsf.unica.it

[b] Scientific Investigation Department (RIS) of Cagliari, Via Ludovico Ariosto, 24, 09129 Cagliari CA (Italy);

[c] Department of Life and Environmental Sciences, University of Cagliari, Via Sant'Ignazio 13, Cagliari (CA), 09123, Italy;

Corresponding author: carlo.ricci@dsf.unica.it; stefania.porcu@dsf.unica.it;

Cannabis samples have been provided from Scientific Investigation Department (RIS) of Cagliari.
A set of 42 samples with different amounts of THC and CBD were studied and in this section are reported the results of GC-FID analysis for both families of samples (THC and CBD rich plants), the optical images and the assignation of vibrational bands that arise form Raman measurements. The samples come from different plantations seized from Scientific Investigation Department (RIS) of Cagliari for the investigation of the content of cannabinoids.

GC-FID analysis

For GC-FID Analysis an Agilent Technologies 7890 series gas chromatograph (GC) was employed, equipped with a split injector and a ULTRA 2 fused silica column: 5% phenyl-methylpolysiloxane, 20 m × 0.32 mm i.d., film thickness 0.52 µm. The analysis has been performed under isothermal conditions at 240°C for 16 min. The injector was maintained at 290 °C. Helium was the carrier gas at 1.4 mL/min; the sample (1 µL) was injected in the split mode (1:50). The GC was fitted with a FID, model 7890B. FID conditions were set as follows: temperature 300 °C; hydrogen was the carrier gas at 40 ml/min and nitrogen the makeup gas at 25 ml/min.

GC-FID analysis has been performed at the Scientific Investigation Department. For the analysis a methanolic solution of each sample has been prepared and androsterone (purchased from Sigma Aldrich) has been used as internal standard.

CBD rich plant

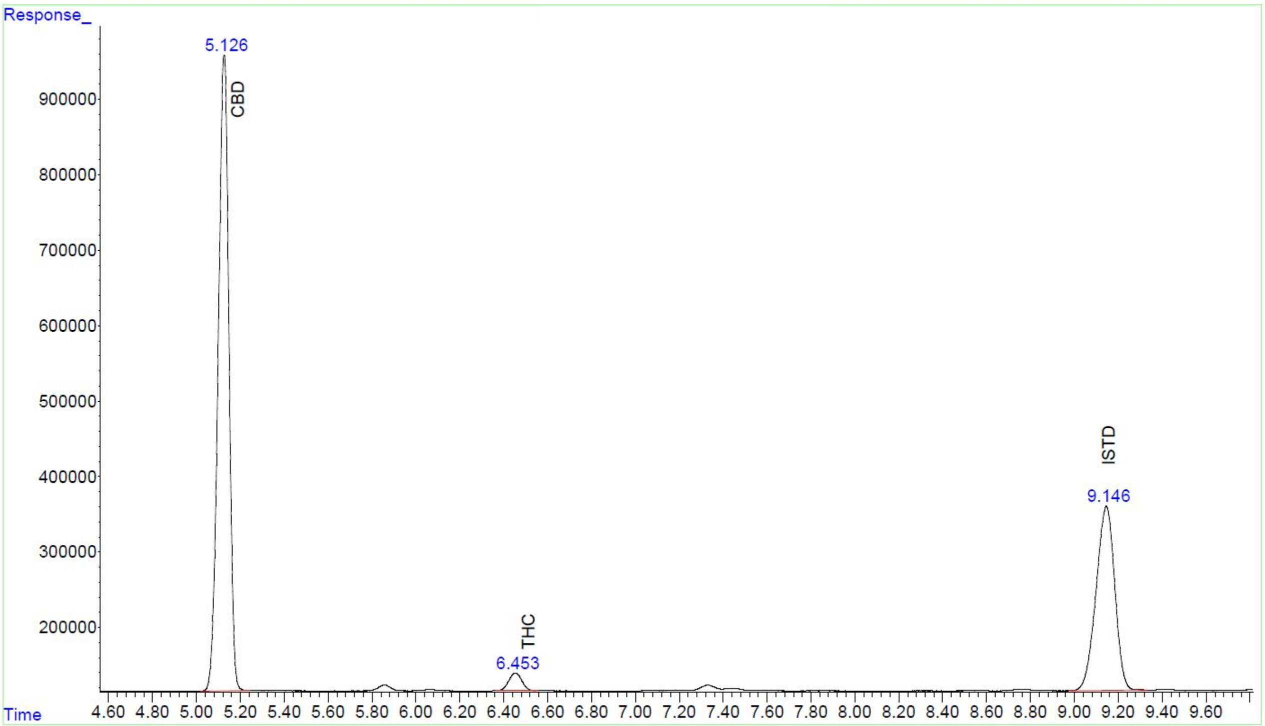

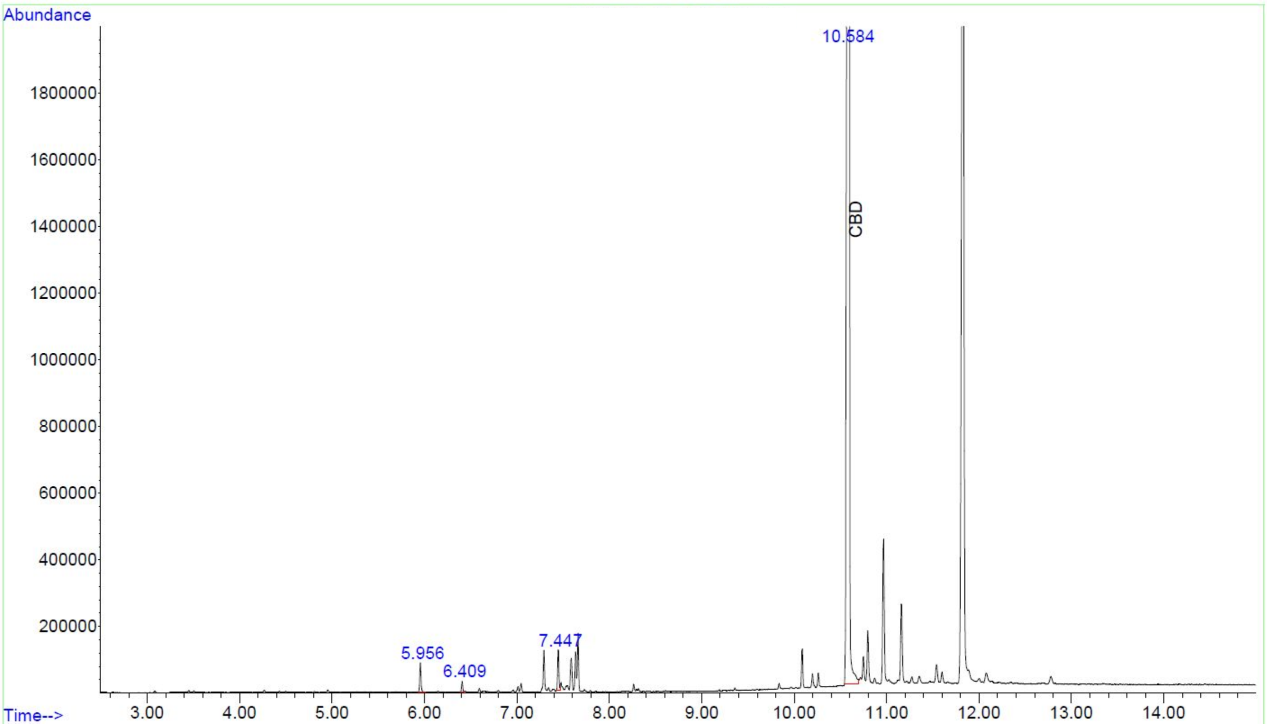

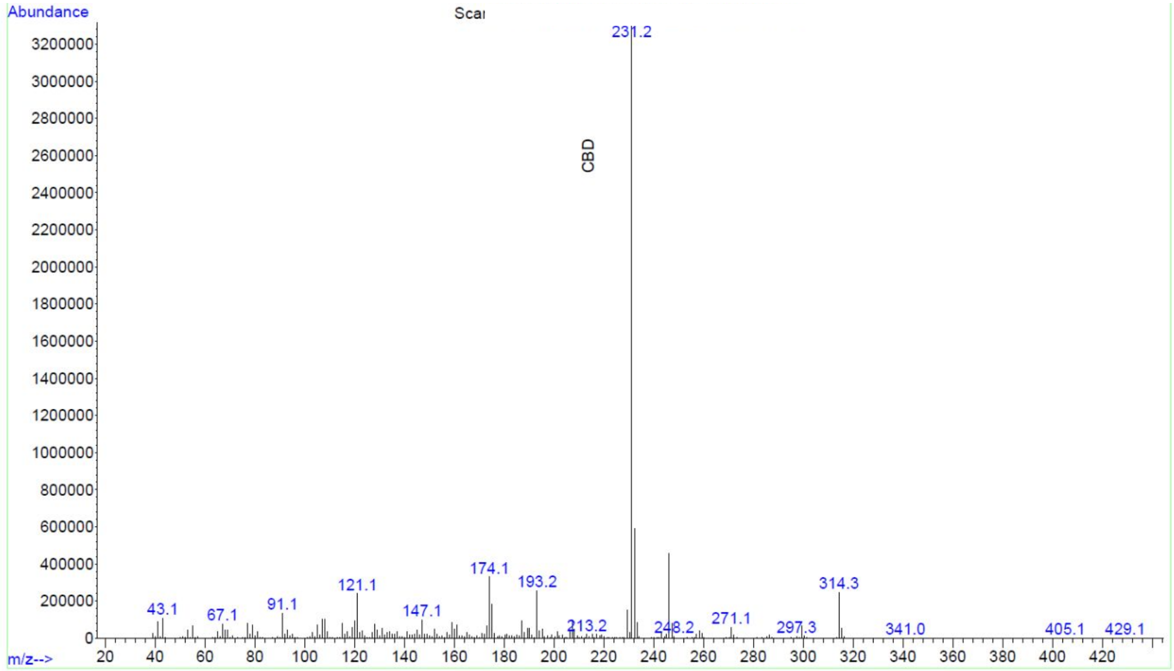

Figure S1. Descriptive chromatogram of CBD rich plants

THC rich plant

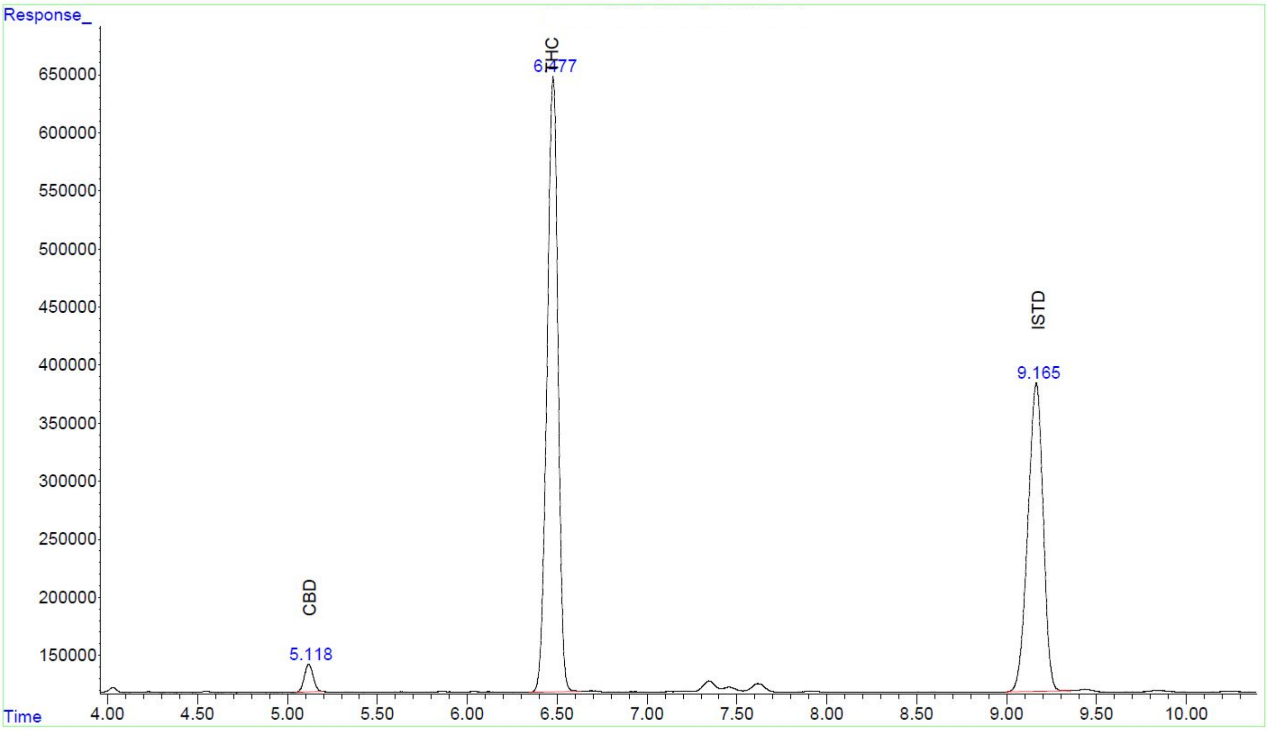

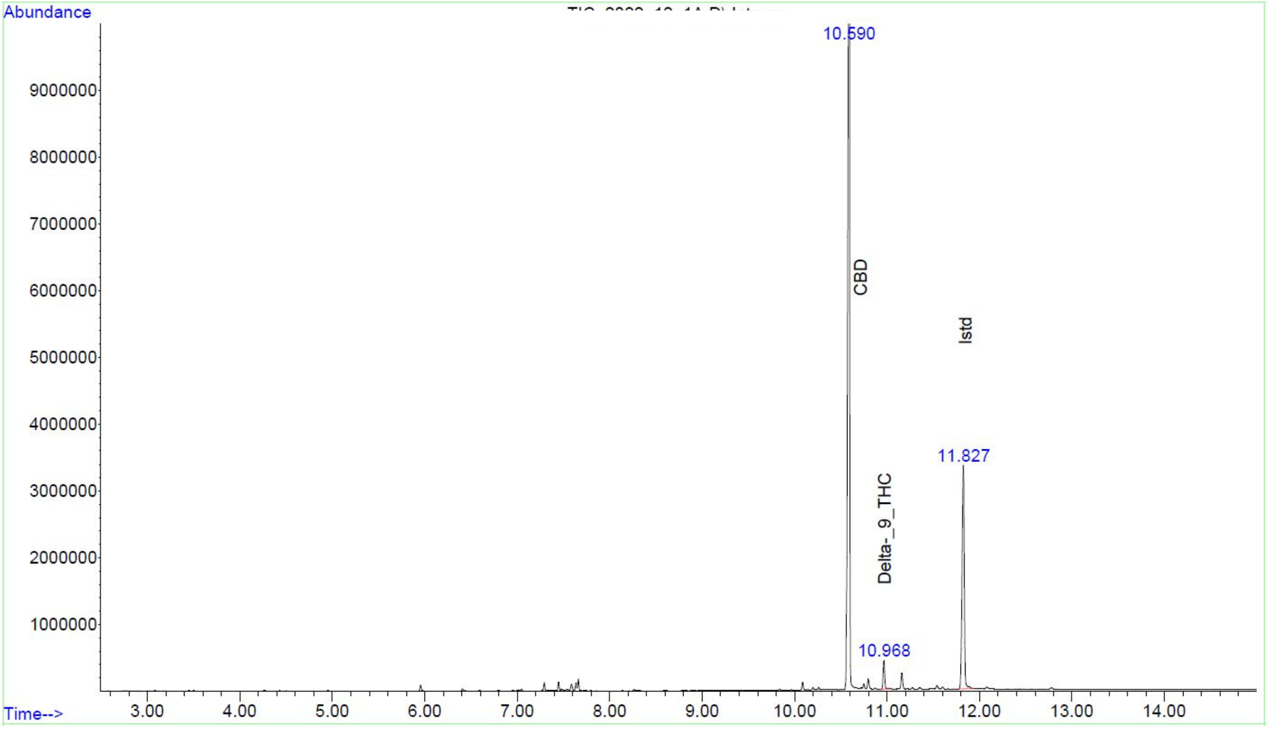

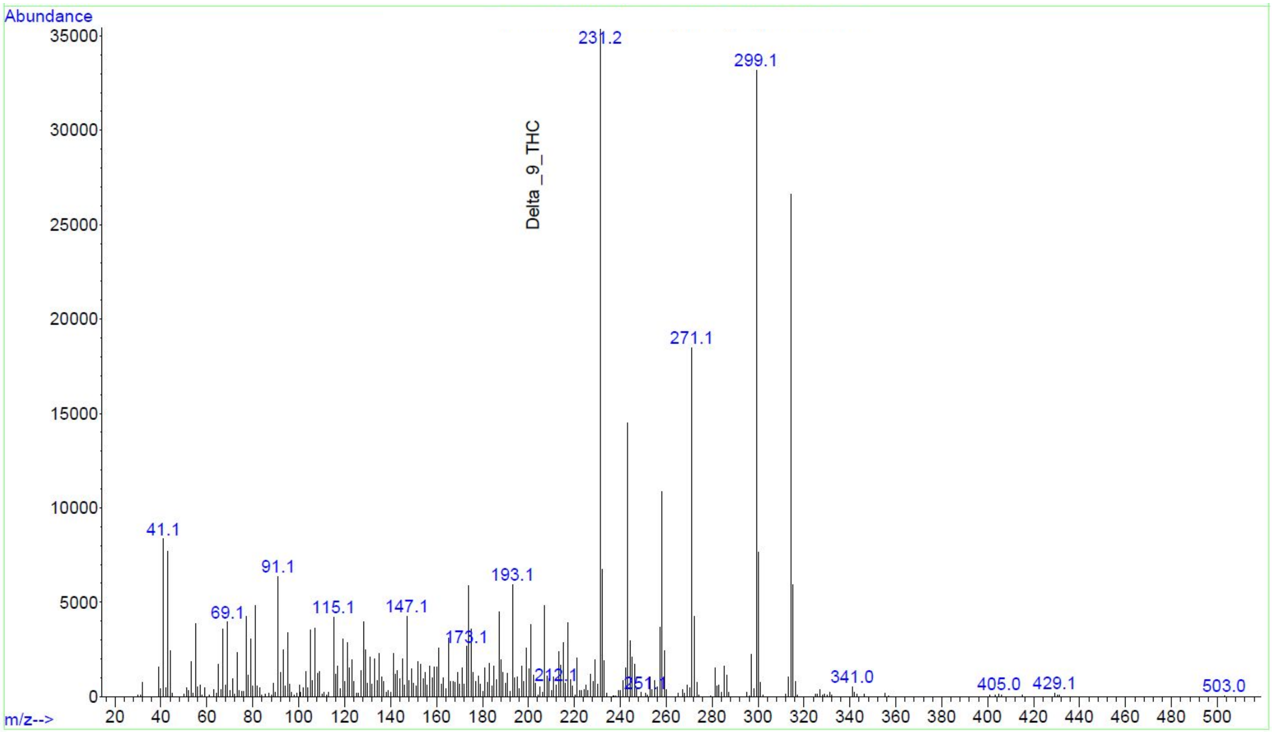

Figure S2. Descriptive chromatogram of THC rich plants

Optical Images

Samples of air-dried *Cannabis sativa* L. bracteal leaves proceedings from inflorescences of both legal and illegal chemotypes were observed by optical microscopy to better interpret Raman microscopy results. A stereo microscope equipped with the HD cam TiEsseLab TrueChrome HD IIS (Tiesselab, Italy) and the software TiEsseLab IS CAPTURE Rel. 3.6.7 (Tiesselab, Italy) was used to take measurements on the structures of interest and to capture the related images

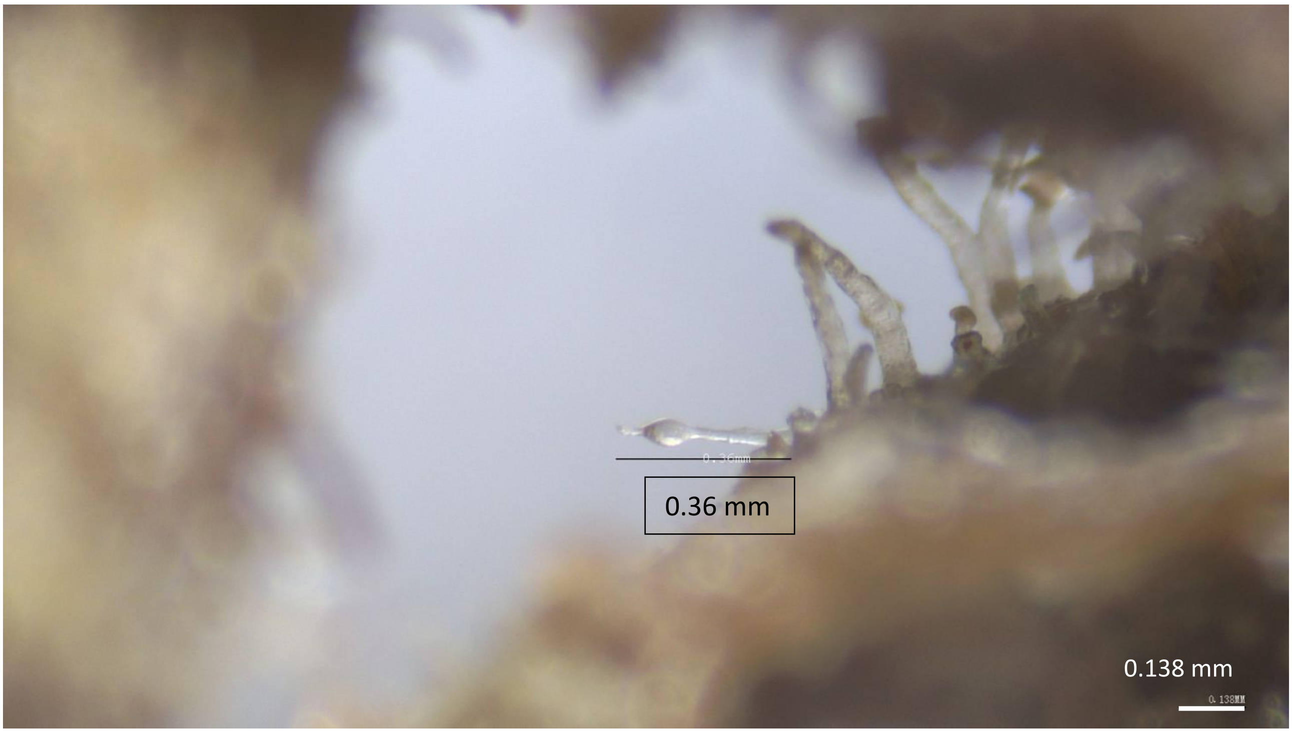

Figure S3 optical image of Sample 6

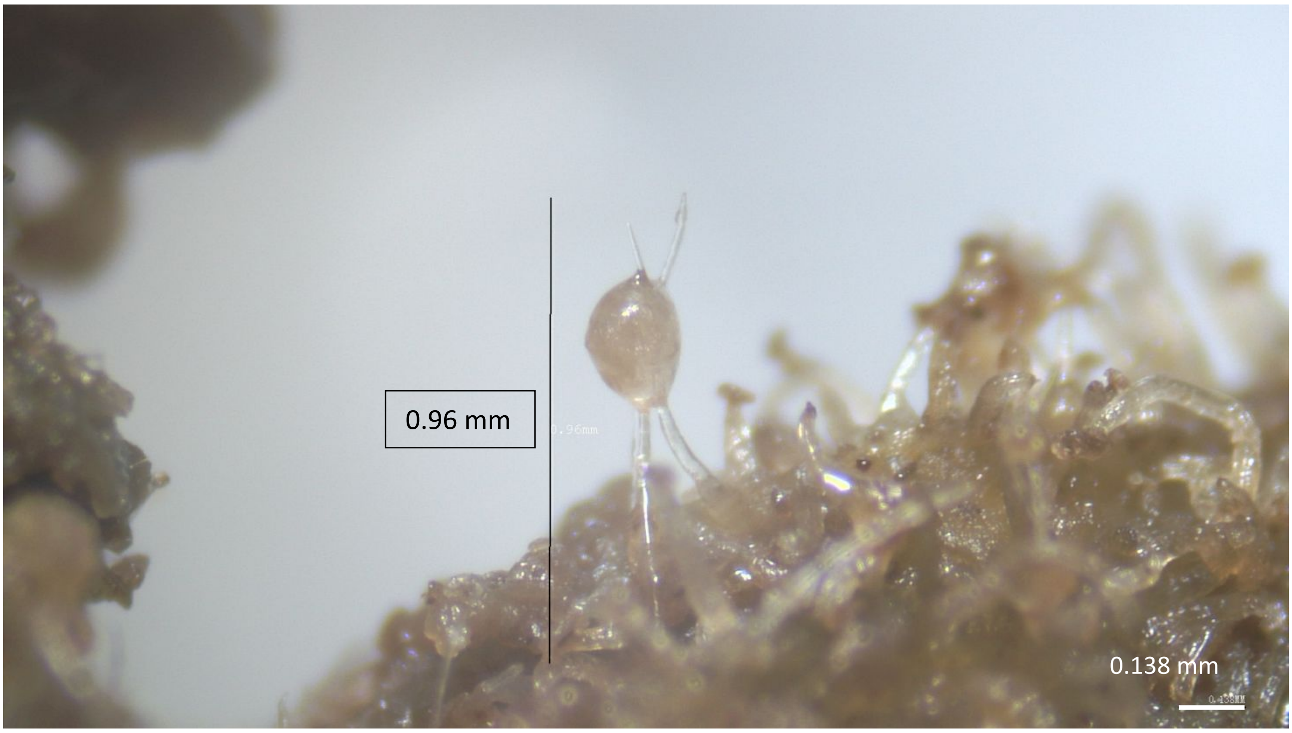

Figure S4 Optical image of Sample 6

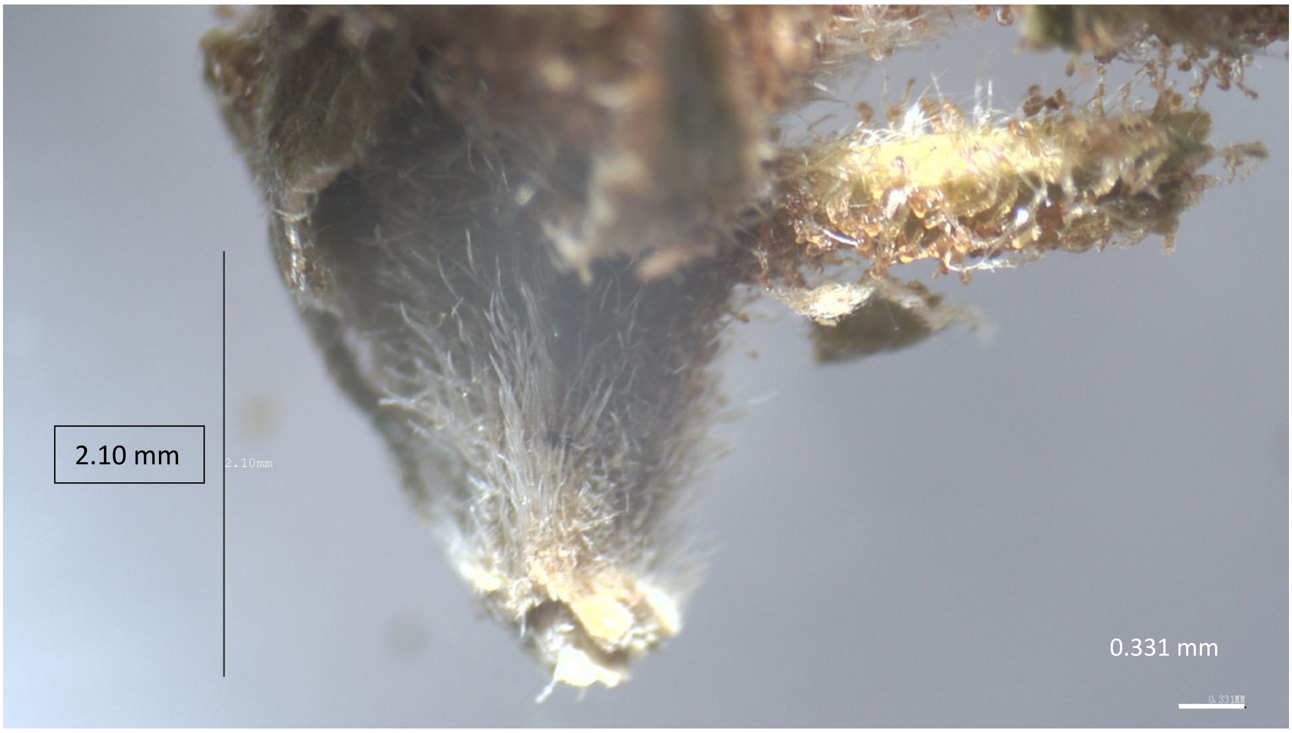

Figure S5 Optical image of Sample 6

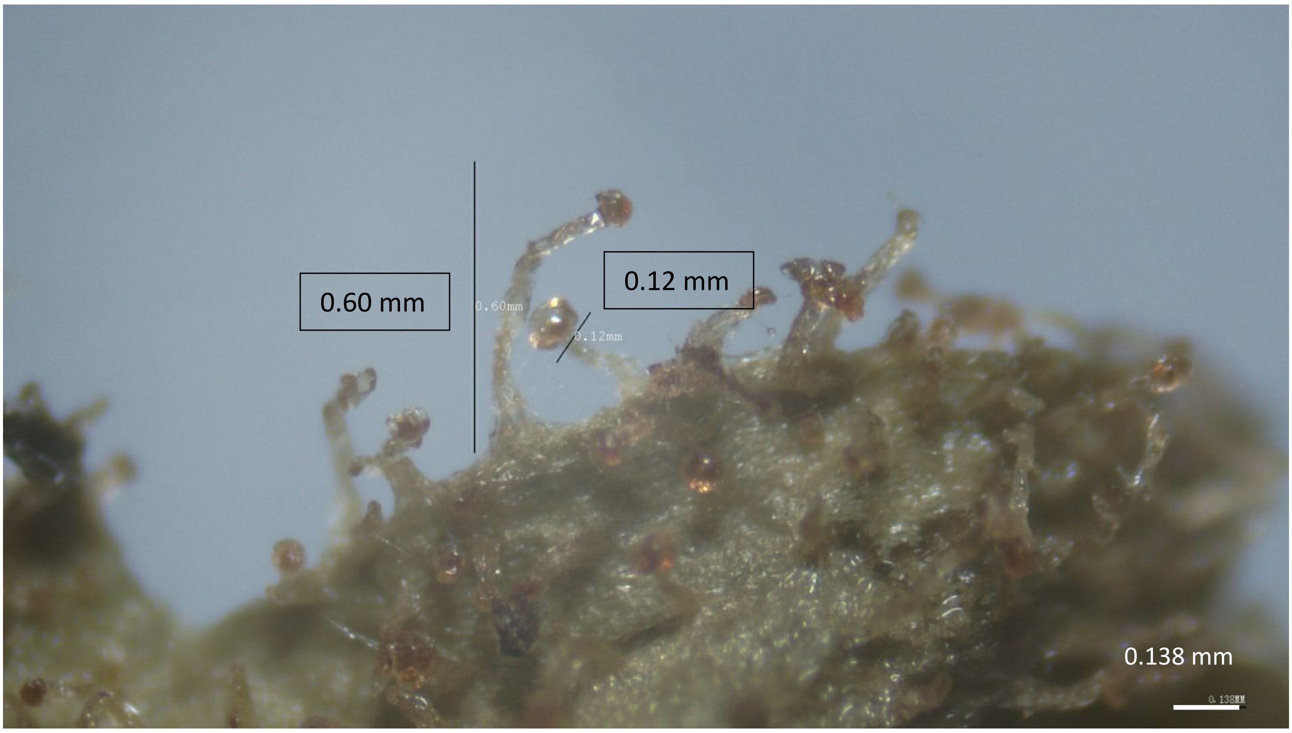

Figure S6 optical image of Sample 26

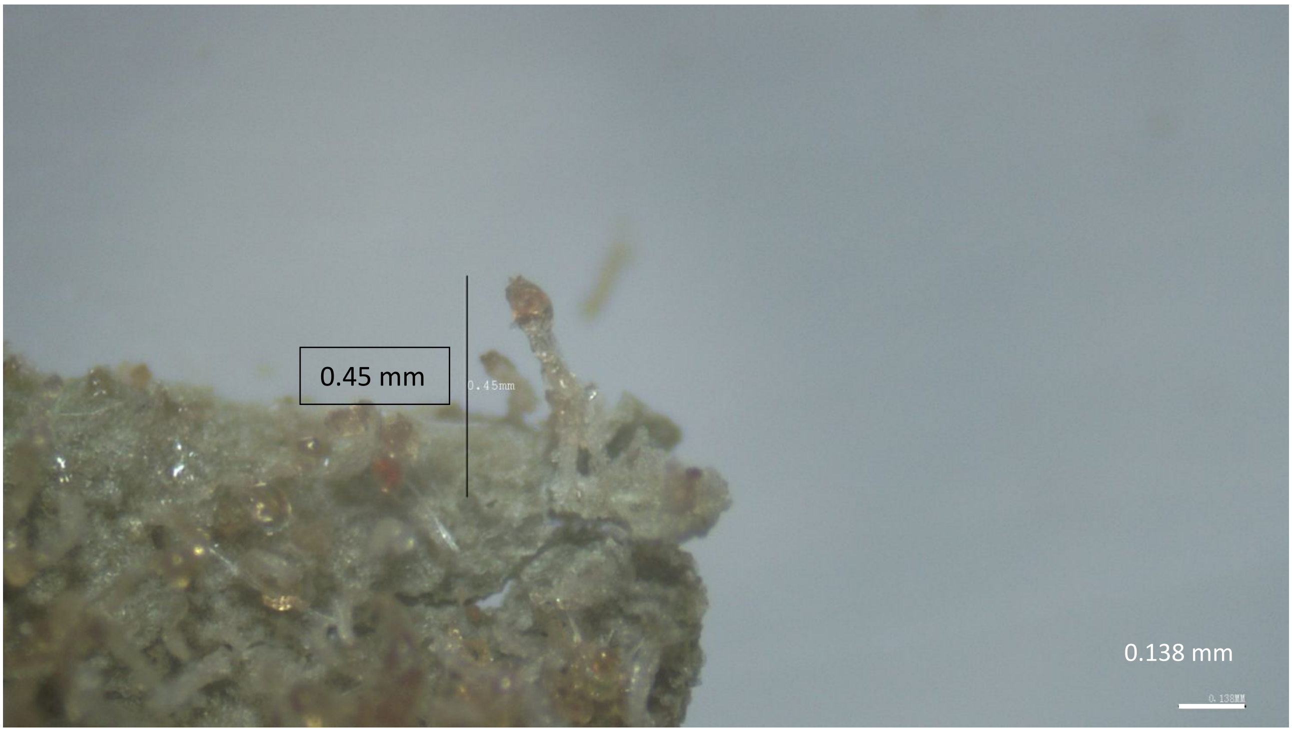

Figure S7 optical image of Sample 26

Assignation of vibrational bands in THC and CBD rich plants

For the Raman measurements, performed at the Physics department of the University of Cagliari, all the samples have been analyzed without previous treatment and the spectra collected from the leaves and inflorescences. For each sample, at least 4 different points were analyzed and the average of them was considered as representative.

The main vibrational bands of the THC rich plant are observed at 775, 780, 835, 1185, 1295, 1321, 1365, 1570, 1600, 1623, 1666 cm^{-1} while, in CBD rich plants vibrational bands at 775, 865, 985,1012, 1080, 1104, 1302, 1340, 1370, 1437, 1643, 1663 cm^{-1} were observed.

Table S1.

**Bands**	**Assignation**
780 cm^{-1}	Cannabinoids
835 cm^{-1}	Cannabinoids
1295 cm^{-1}	bending vibration C−C−H of the aliphatic chain in Cannabinoids (THC)
1301 cm^{-1}	Cannabinoids
1440 cm^{-1}	δ(CH2) + δ(CH3) vibration of the aliphatic chain in CBD
1623-1666 cm^{-1}	Arimatics vibration in Cannabinoids
